# Development of Central Nervous System Vasculitis in a Patient with Waldenstrom Macroglobulinemia: A Rare Presentation with Poor Prognosis

**DOI:** 10.7759/cureus.6039

**Published:** 2019-10-30

**Authors:** Tahir Muhammad Abdullah Khan, Yusra Ansari, Abdul Hasan Siddiqui, Saad Ali Ansari, Faraz Siddiqui

**Affiliations:** 1 Internal Medicine, Marshfield Medical Center, Marshfield, USA; 2 Internal Medicine, Rawalpindi Medical College, Rawalpindi, PAK; 3 Pulmonary and Critical Care Medicine, Staten Island University Hospital / Northwell Health, Staten Island, USA; 4 Internal Medicine, Pakistan Institute of Medical Sciences, Islamabad, PAK; 5 Pulmonary and Critical Care Medicine, Robert Packer Hospital, Sayre, USA

**Keywords:** cns vasculitis, waldenström macroglobulinemia, cryoglobulinemia

## Abstract

Waldenstrom macroglobulinemia (WM) is a rare lymphoproliferative disorder characterized by the presence of monoclonal immunoglobulin M in serum. WM may present with neurologic complications involving the peripheral and central nervous systems (CNS) though CNS complications associated with WM are rare. We present a case of a 72-year-old male with an 18-month history of WM who experienced neurologic and constitutional symptoms indicative of WM progression over a three-week period while on rituximab maintenance therapy. The patient’s symptoms were initially attributed to rituximab-induced asthenia though his clinical condition did not improve with rituximab discontinuation. Due to progressively worsening neurologic symptoms, the patient was re-evaluated and found to have multiple cerebral infarcts and increased serum cryoglobulin levels indicative of cryoglobulinema. The patient was promptly initiated on a combination regimen of high dose steroids, intravenous immunoglobulin (IVIG), and plasmapheresis but had a poor response. Brain biopsy revealed necrotizing vasculitis with dense intra- and peri-vascular CD3 positive T-cell infiltrates with mural necrosis. This is a unique case of WM complicated by type 1 cryoglobulinemia associated with CNS vasculitis that was unresponsive to active rituximab therapy; this case illustrates a poor prognosis of patients with CNS involvement in WM.

## Introduction

Waldenstrom macroglobulinemia (WM) is a rare lymphoproliferative disorder characterized by the presence of serum monoclonal immunoglobin M (IgM) proteins associated with ≥10% of clonal lymphoplasmacytic cells infiltrating the bone marrow [1-2]. The disease may present with signs or symptoms secondary to either direct lymphohematopoeitc organ system infiltration or as a sequelae of bone marrow infiltration i.e., anemia and/or thrombocytopenia [2-8]. Patients can also present with hyperviscosity syndrome and neurologic complications with peripheral neuropathy being more common compared to other neurologic sequelae [6-10]. Even though central nervous system (CNS) involvement associated with WM is rarely reported in the literature, it may confer a poor prognosis to disease [6, 8-9, 11-12]. We present a case of a 72-year-old man with WM who developed CNS vasculitis complicated by cryoglobulinemia while on concurrent maintenance therapy with rituximab. This case highlights poor prognosis for patients with WM complicated by CNS vasculitis.

## Case presentation

**Figure 1:**
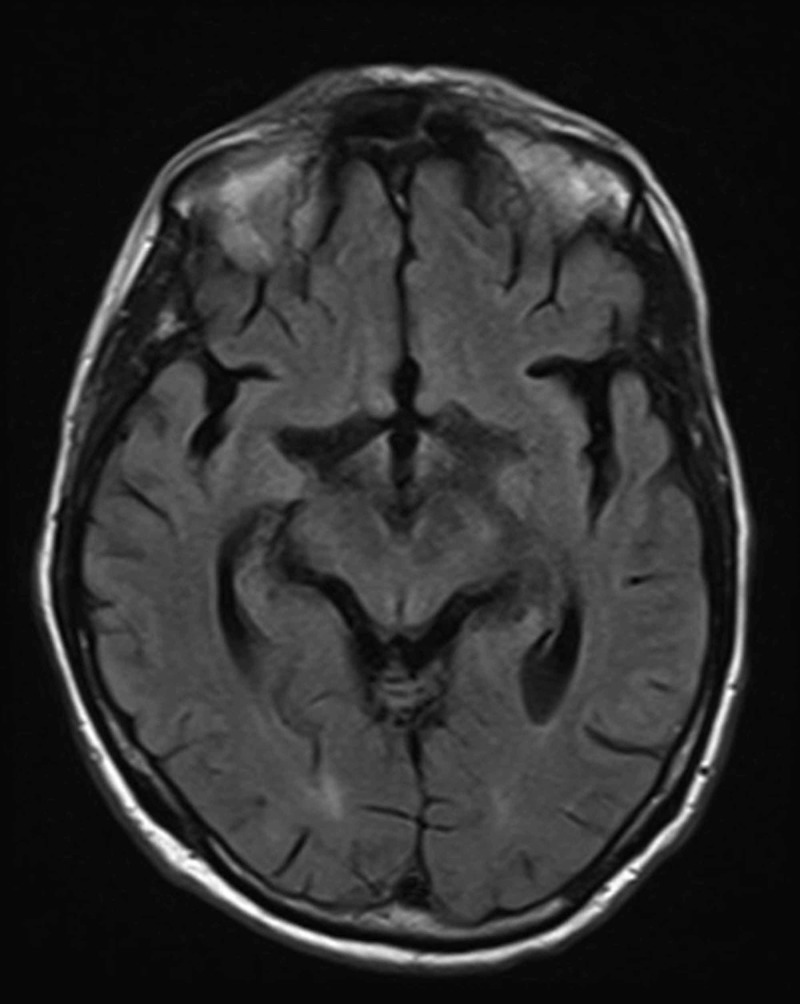
T2-weighted MRI of brain. T2-weighted MRI of brain revealing no acute intracranial pathology.

A 72-year-old male with an approximately 18-month history of lymphoplasmacytic lymphoma (LPL) specifically WM with bone marrow involvement gradually developed nonspecific symptoms of worsening memory, generalized weakness, malaise, fatigue, intermittent dizziness, and loss of appetite over a three-week period. The patient had previously undergone successful induction therapy with six cycles of bendamustine and rituximab and was currently on maintenance therapy with rituximab. Other medical history of note included hypertension, dyslipidemia, and probable polymyalgia rheumatica on treatment with low-dose systemic prednisone therapy. Until he visited the clinic one week prior to hospitalization, the patient had clear mentation with no signs of confusion; however, he reported experiencing worsening fatigue, generalized weakness, forgetfulness, and intermittent dizziness that he described as feeling 'woozy' and had increased sleepiness during the day. The MRI of the brain revealed no acute abnormality to explain the symptoms (Figure 1). Due to lack of significant deficits detected during a complete neurologic examination and no abnormalities on brain MRI (Figure 1), it was assumed that rituximab-induced asthenia was contributing to symptoms; thus, the last scheduled dose of rituximab was held.

**Figure 2:**
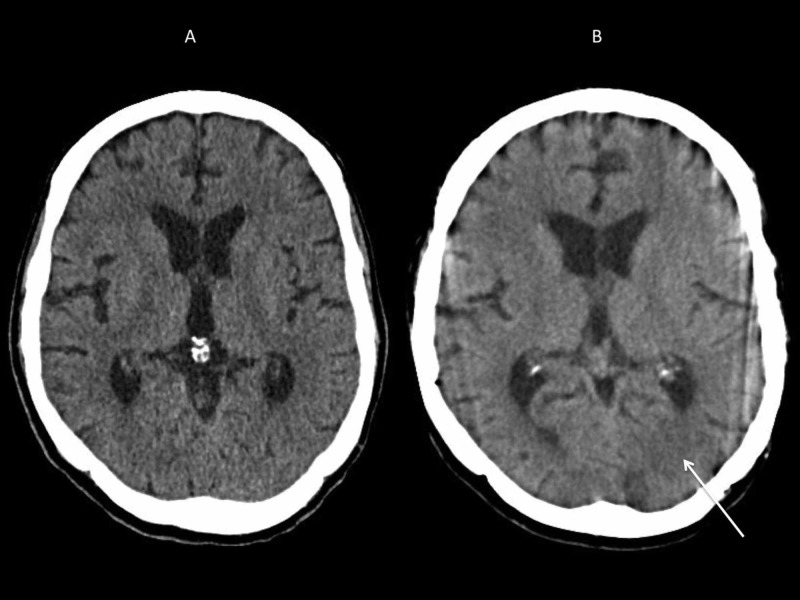
CT scan of brain on the day of admission (A) and at 48 hours after admission (B). A: CT brain performed on the day of hospitalization showed no acute intracranial pathology. B: CT brain performed at 48 hours after hospitalization showed focal abnormalities in the left parieto-occipital areas of brain (arrow).

The patient’s clinical condition continued to deteriorate, and he experienced new onset of symmetrical bilateral hand numbness and tingling associated with worsening of weakness and dizziness one week after the initial clinic visit which prompted hospitalization for further evaluation. On the day of admission, an initial complete physical examination was normal except for bilateral mild paresthesias of the hands reported by the patient. Initial lab work including complete blood count, comprehensive metabolic panel, and urinalysis were normal, and a CT scan of the brain revealed no acute intracranial abnormality (Figure 2A). Within 48 hours of hospitalization, the patient developed confusion without focal neurological deficit. A follow-up CT of the brain showed subtle low-density regions in the left frontal, parieto-occipital, and right temporal lobe regions (Figure 2B).

**Figure 3:**
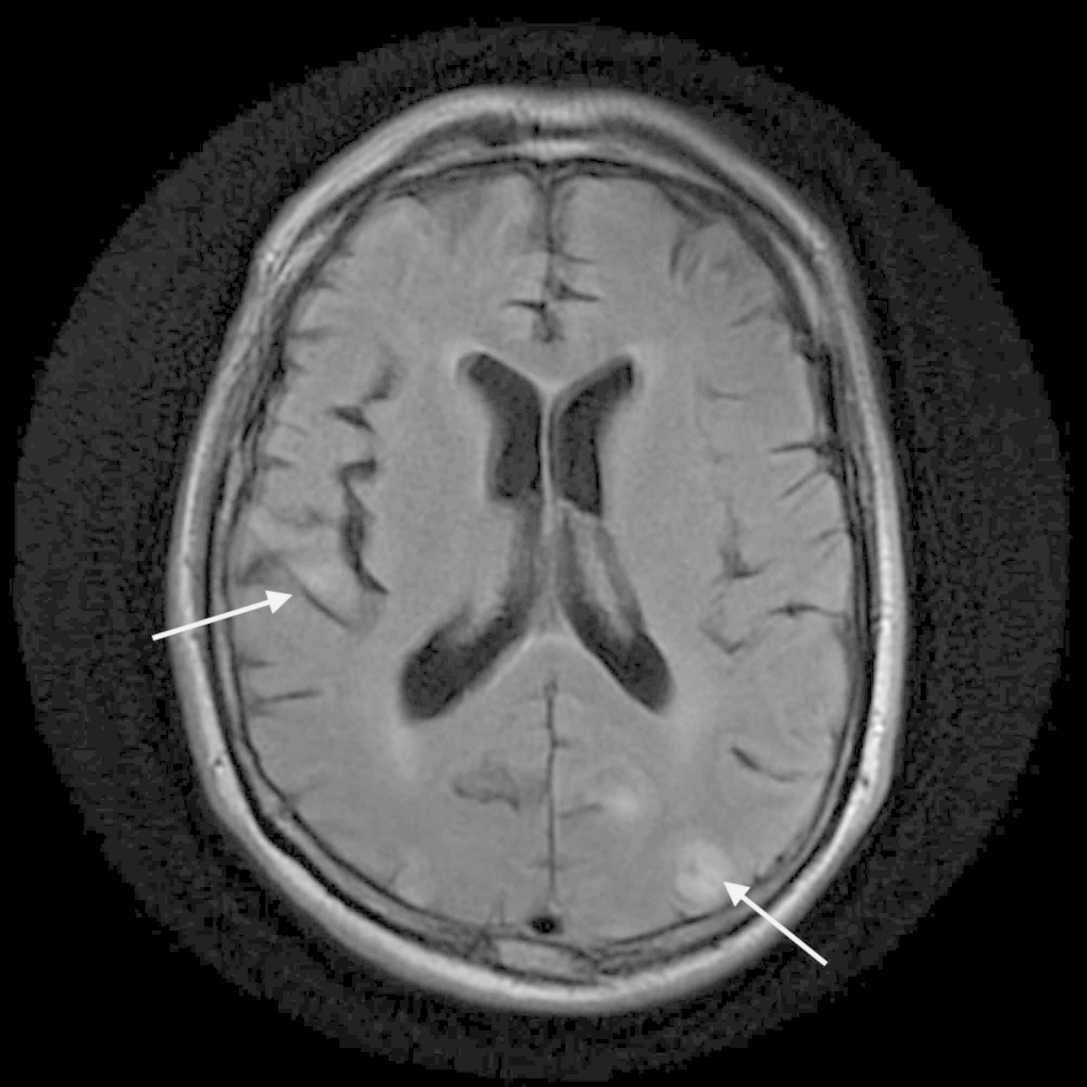
T2 FLAIR MRI of the brain with and without contrast. T2 fluid attenuated inversion recovery (FLAIR) MRI of the brain showed new multifocal areas of abnormal FLAIR hyperintensity signals in the supratentorial cortices and subcortical white matter (arrows).

Subsequent brain MRI with and without contrast revealed new multifocal areas of abnormal fluid attenuated inversion recovery (FLAIR) hyperintensity signals in the supratentorial cortices and subcortical white matter with accompanying possible patchy leptomeningeal enhancement (Figure 3). However, accompanying diffusion weighted imaging (DWI) revealed no increased fluid signal motion or overt hemosiderin staining.

Combined, these imaging results were suggestive of progressive multifocal leukoencephalopathy versus atypical presentation of posterior reversible encephalopathy syndrome with a less likely subacute infarction as the recent brain MRI did not detect any ischemic lesions. Due to history of WM, hyperviscosity syndrome was excluded by serum hyperviscosity testing, and serum protein electrophoresis revealed elevated levels of monoclonal IgM (2,630 mg/dL) which was improved since the patient’s last reading of 3,190 mg/dL three months prior to neurologic symptom onset. The CNS involvement of lymphoma, paraneoplastic, and infectious causes were excluded by cerebrospinal fluid (CSF) analysis for infections, malignancy, and paraneoplastic antibodies. Hypothyroidism and cyanocobalamin deficiency were ruled out with normal endocrine testing; patient had normal C-reactive protein (CRP) and procalcitonin levels but had an elevated erythrocyte sedimentation rate (ESR) of 77 mm/hour. To determine whether CNS vasculitis was contributing to current symptoms, additional laboratory testing was performed which was positive for cryoglobulin yet with normal C3 complement levels. Further infectious disease work-up was negative for HIV, hepatitis, Lyme and anaplasmosis, blastomycosis, cryptococcal antigen, John Cunningham (JC) polyoma DNA virus, herpes simplex virus 1 and 2, varicella-zoster virus, Epstein-Barr virus, cytomegalovirus, enterovirus, and arbovirus. Based on clinical presentation and findings from brain imaging and laboratory testing, it was assumed that cryoglobulinemia was most likely causing CNS vasculitis; therefore, the patient was initiated on a regimen of high-dose systemic steroids followed by intravenous immunoglobulin (IVIG), and plasma exchange therapy.

**Figure 4:**
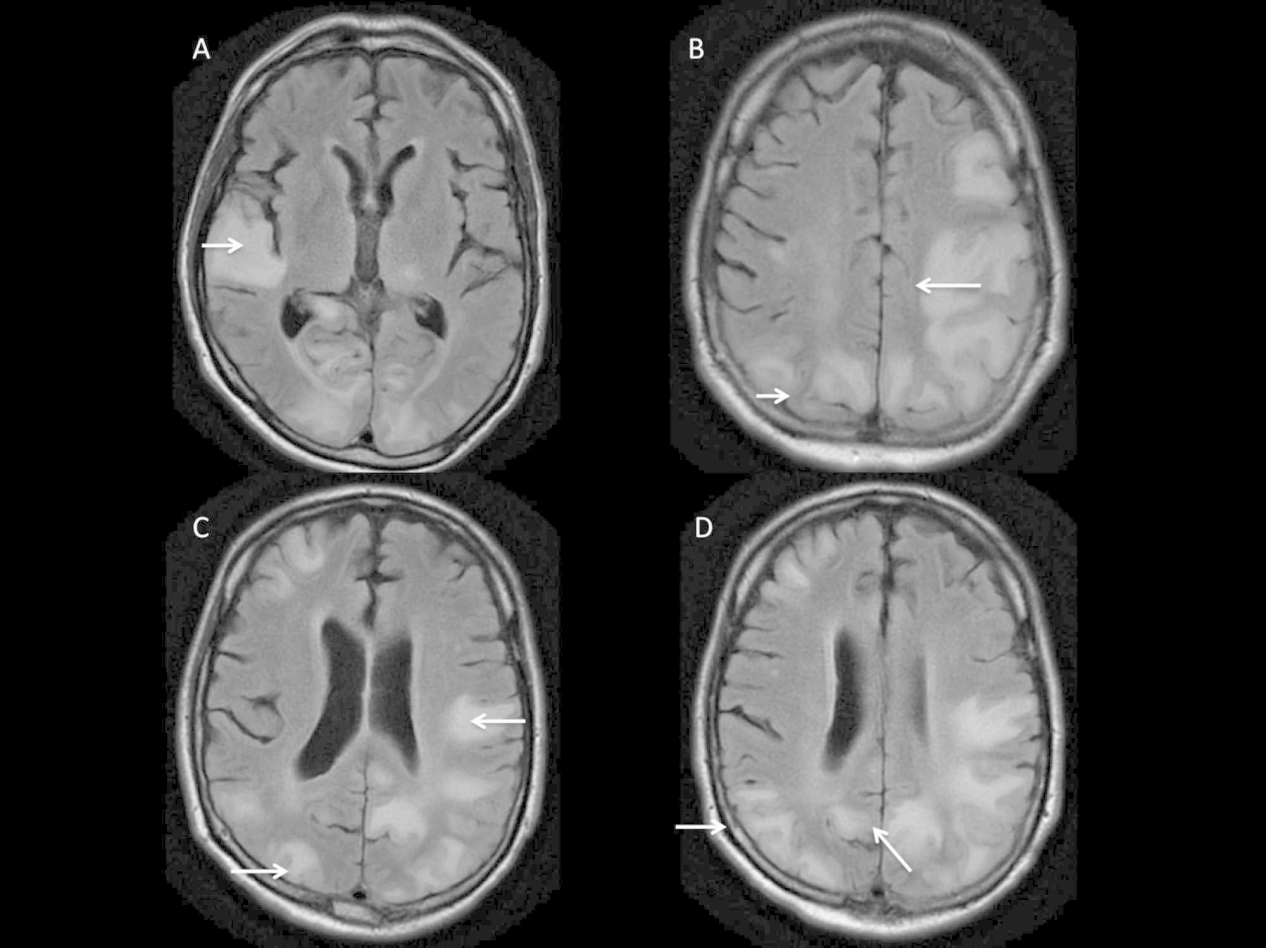
T2 - FLAIR MRI of brain. MRI of brain (A-D) demonstrates the development of new multifocal areas of enhancement (arrows) suggestive of new ischemic lesions in multiple vascular territories.

Unfortunately, despite high dose systemic steroids followed by five sessions of plasma exchange treatment and IVIG therapy, the patient’s neurologic status rapidly declined, and he experienced severe encephalopathy with cortical blindness complicated by seizure activity. A serial MRI of the brain (Figure 4) showed worsening lesions in both cerebral hemispheres, thalamus, and cerebellum areas though no restricted diffusion or enhancement with relatively rapid radiologic changes indicated against progressive multifocal leukoencephalopathy (PML); however, progressive reversible encephalopathy syndrome (PRES) remained a consideration.

Due to progressive neuro-radiologic changes and poor response to empiric therapy with unclear diagnosis, a brain biopsy was pursued that revealed dense micro-gliosis and astrogliosis with frequent bizarre astrocytes with groundglass oligodendrocytes. A comprehensive infectious disease work-up was negative including immunostains and in situ hybridization for JC virus. Additional pathology analysis detected multiple foci of necrotizing vasculitis with dense intra- and peri-vascular CD3 positive T-cell infiltrates with mural necrosis associated with dense reactive gliosis of both gray-white matter and fibrin deposition in sub-pial gray matter and in the perivascular spaces adjacent to necrotizing leukocytoclastic vasculitis which indicated diffuse acute and subacute ischemic infarction. No evidence of active demyelinating process or amyloid beta-related angiopathy was detected. Brain tissue biopsy was also negative for CD 20 positive cells as well as for any malignant or infectious process.

The patient’s hospital course was further complicated by respiratory distress and acute hypoxic respiratory failure secondary to aspiration pneumonia requiring mechanical ventilation which was successfully treated with broad-spectrum antibiotics, and the patient was weaned off from the mechanical ventilator. However, the patient did not recover from neurologic injury and continued to have severe encephalopathy that progressed to coma. Due to poor prognosis, the patient’s family opted for strict comfort care where the patient later died.

## Discussion

Lymphoplasmacytic lymphoma, previously known as lymphoplasmacytoid lymphoma, is a low-grade mature B-cell non-Hodgkin lymphoma that produces immunoglobulins and usually involves the bone marrow with less common involvement of the spleen, lymph nodes, and/or other sites [2-4, 6-8, 13]. The MYD88 L265P mutation is present in 90% of patients with WM though this mutation is not specific for WM, as it is expressed in approximately 50% of patients with monoclonal gammopathy of undetermined significance (MGUS) IgM [2, 13]. Lymphoplasmacytic neoplasms can also produce other immunoglobulins in addition to IgM including IgG, IgA, mixed cryoglobulin, and gamma heavy chains [2, 4, 6-7, 14-15]. WM is a type of LPL that produces monoclonal IgM which confers its name [2, 6-8]. Age-adjusted incidence rates indicate that WM is a rare condition in patients <50 years with sharp increases in incidence for both genders in older demographics; WM is slightly more common in males than in females [1-5, 16].

Waldenstrom macroglobulinemia is generally diagnosed by elevated serum monoclonal IgM protein in addition to more than 10% of bone marrow infiltrated with lymphocytes characteristic of lymphoplasmacytoid cells [1-2] . Abnormal levels of proteins such as cryoglobulins, cold agglutinins, monoclonal kappa and lambda light chains, immunoglobulins, and Bence Jones protein, may also be present [1-7, 11-12, 14-15]. Other laboratory abnormalities associated with WM include cytopenias, decreased hemoglobin levels, decreased levels of serum albumin, elevated lactate dehydrogenase (LDH) levels, and elevated beta-2 microglobulin levels [1-8, 12, 14]. WM may present with a wide array of clinical symptoms, and the presenting symptoms vary based upon numerous factors and can manifest by either direct tumor infiltration of hematopoietic and lymphatic organ system such as anemia, thrombocytopenia, enlarged lymph nodes, and hepatosplenomegaly or as a sequelae to abnormal monoclonal IgM production resulting in hyperviscosity syndrome or cryoglobulinemia causing peripheral neuropathy and/or inflammation of small vessels [1-8, 10-12, 15]. However, many patients with WM can be asymptompatic [1-4, 6-8, 14]. In the case series by García-Sanz et al., 27% of patients with WM were asymptomatic at time of diagnosis, and in patients with WM, serum hyperviscosity and high beta-2 microglobulin levels were identified as predictors of poor overall survival [3]. In a recent survey of the WM-associated literature, Gertz determined that in addition to monoclonal IgM and beta 2 microglobulin levels, patient age, platelet count, and hemoglobin level are also predictive for adverse outcomes in patients with WM [2].

According to case series by García-Sanz and Levine, neurologic manifestations of WM are common, occurring in 22% and 48% of the studied patient populations, respectively, and predominantly present as peripheral neuropathy involving mostly the sensory nerves [3, 6-7, 9-10]. Other neurologic manifestations include mononeuritis multiplex, multifocal motor neuropathy, cranial nerve palsies, and multifocal leukoencephalopathy as seen in patients with Bing-Neel syndrome [6, 9]. The CNS involvement may occur mostly as sequelae of hyperviscosity syndrome when the patient's serum viscosity reaches ~4-5 cP (centipoise) which corresponds to our patient’s serum IgM level of at least 3 g/dL, and usually manifests as bleeding, visual disturbances, and headaches or other neurologic symptoms [2, 6-9, 17-19]. CNS vasculitis in patients with WM is very rarely reported though vasculitis development is possible due to the presence of abnormal levels of IgM and/or corresponding establishment of immune complexes in these patients [6-9, 11-12, 15, 18, 20]. Our patient developed CNS vasculitis associated with WM by producing monoclonal IgM with cryoglobulin activity.

Cryoglobulinemia is diagnosed when blood proteins precipitate at temperatures <37°C [19]. Patients with cryoglobulinemia usually present with skin lesions including purpura and Raynaud’s phenomenon, arthralgia, peripheral neuropathy, and glomerulonephritis though our patient had no current or previous history of dermatologic manifestations of this condition [18-19]. Cryoglobulinemia is associated with various underlying diseases including chronic hepatitis C infection, disorders with monoclonal gammopathy [WM, monoclonal gammopathy of undetermined significance (MGUS), and multiple myeloma (MM)], and autoimmune disease such as systemic lupus erythematosis (SLE) and Sjögren's syndrome [18]. Cryoglobulinemic vasculitis usually affects small- to moderate-sized vessels via immune complex-mediated damage and is generally diagnosed by testing for cryoglobulin in the serum and histologic findings on biopsy [18]. In contrast, leukocytoclastic vasculitis is a small vessel inflammatory disease usually associated with certain medications, infections, malignancies, and collagen vascular diseases [20]. Prognosis of vasculitis varies according to the underlying disease and the management is based on treating the underlying cause [18, 20].

We have described a rarely reported case of WM with development of type I cryoglobulinemia complicated by CNS leukocytoclastic vasculitis while on maintenance rituximab therapy for six months after finishing six cycles of induction therapy with bendamustine and rituximab. Even though monoclonal IgM levels consistently decreased during maintenance rituximab therapy, our patient developed cryoglobulin proteins which probably contributed to the development of CNS leukocytoclastic vasculitis. To the best of our knowledge, cryoglobulinemia-associated leukocytoclastic CNS vasculitis development in a patient with WM while on active rituximab therapy has not been reported in the literature though Riangwiwat et al. (2016) described the diagnosis and treatment of a female patient who developed WM-associated CNS vasculitis post-rituximab and thalidomide maintence therapy, and Arjunan and Rai reported the diagnosis and treatment of a male patient with WM-associated CNS involvement that was refractive to antibody-based therapies including rituximab [11-12]. In contrast to Riangwiwat et al. and similar to Arjunan and Rai, our patient did not respond to intravenous steroids or IVIG and plasmapheresis and continued to experience neurologic decline though cryoglobulinemic vasculitis is generally managed by treatment of the underlying disorder and, in emergency situations, plasma exchange therapy [11-12, 18]. However, it is uncertain whether rituximab or the underlying B-cell malignancy is implicated in transformation of the patient’s WM to result in insidious onset of cryoglobulinemia and leukocytoclastic vasculitis with CNS involvement though we propose rituximab therapy may have contributed to the aggressive behavior of the disease with resistance of CNS leukocytoclastic vasculitis to standard plasma exchange and high dose steroid regimen based on the timing and progression of our patient’s symptoms. Furthermore, Mauermann et al. has previously described a patient with WM who developed an unusual transition of type I cryoglobulinemia to type II cryoglobulinemia while on rituximab treatment and subsequently developed mononeuritis multiplex in addition to other symptoms of systemic vasculitis [15]. Similarly, rituximab therapy has previously been implicated in causing worsening of neuropathy symptoms as well as progressive multifocal leukoencephalitis in patients with WM [7, 9, 15]. We assume that the development of cryoglobulinemia-associated CNS vasculitis in patients with WM treated with rituximab carries a poor prognosis as observed in our patient. However, more studies are needed to further elucidate whether rituximab is implicated in the pathogenesis of aggressive leukocytoclastic vasculitis of CNS or its possible role in changing the behavior of WM when rituximab is continued for long-term maintenance treatment. A high index of suspicion should be kept to promptly initiate diagnostic work-up and treatment of patients with WM who develop symptoms consistent with CNS vasculitis to improve survival though it is unknown whether the currently available treatments of plasmapheresis and high dose steroids are effective in treating this complication of WM.

## Conclusions

We presented a rare but fatal case of WM with predominant cryoglobulinemia-associated CNS leukocytoclastic vasculitis that was nonresponsive to standard therapy of high intensity steroids and plasmapheresis post-rituximab therapy. Additional work is needed to examine the underlying pathophysiology of CNS vasculitis associated with WM and develop novel treatments for this ominous complication. Furthermore, the role of long-term rituximab therapy in the pathogenesis of CNS leukocytoclastic vasculitis in patients with WM needs to be elucidated.
